# Supplementary figures and images for: Phosphatidylserine regulates plasma membrane repair through tetraspanin-enriched macrodomains
Source: J Cell Biol. 2024 Mar 26;223(6):e202307041. doi: 10.1083/jcb.202307041 (PMC10964951; doi:10.1083/jcb.202307041)

ORP9

GAPDH

blot

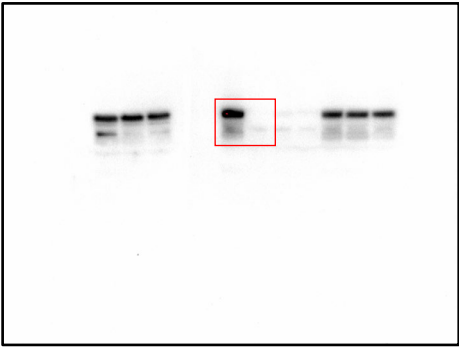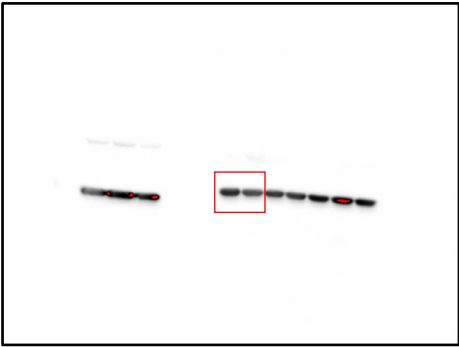

ladder

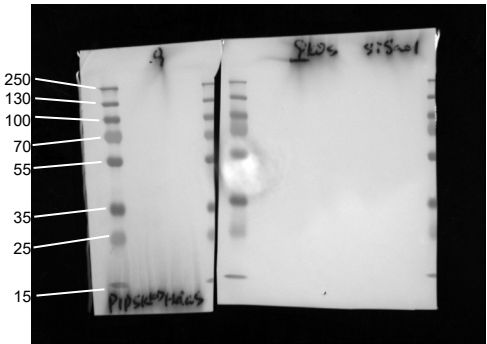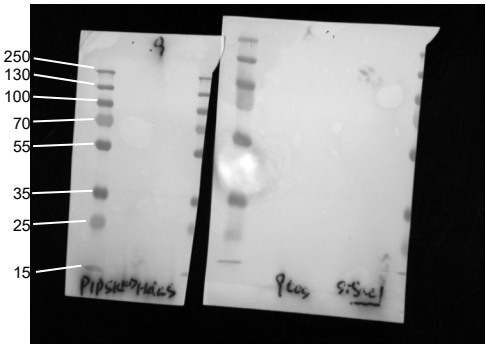

merge

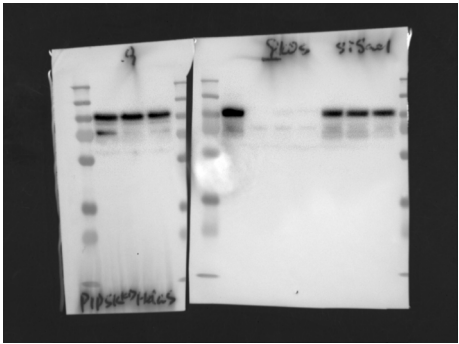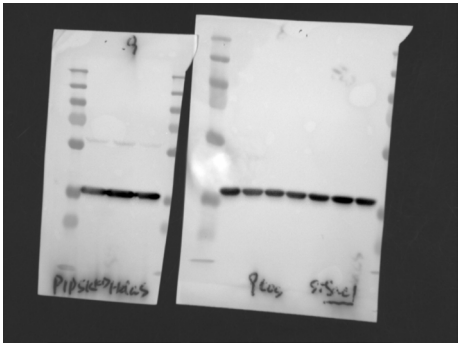

Supplement: SourceData F2 — is the source file for Fig. 2. [file JCB_202307041_SourceDataF2.pdf]
